# Vascular risk factors for male and female urgency urinary incontinence at age 68 years from a British birth cohort study

**DOI:** 10.1111/bju.14137

**Published:** 2018-03-07

**Authors:** Alex Tsui, Diana Kuh, Linda Cardozo, Daniel Davis

**Affiliations:** ^1^ MRC Unit for Lifelong Health and Ageing at UCL London UK; ^2^ Department of Urogynaecology King's College Hospital London UK

**Keywords:** urgency, urinary incontinence, vascular, adiposity, prevalence, ageing, #incontinence

## Abstract

**Objective:**

To investigate the prevalence of urgency urinary incontinence (UUI) at age 68 years and the contribution of vascular risk factors to male and female UUI pathogenesis in addition to the associations with raised body mass index (BMI).

**Subjects and methods:**

In all, 1 762 participants from the Medical Research Council (MRC) National Survey for Health and Development birth cohort who answered the International Consultation on Incontinence Questionnaire short form (ICIQ‐SF), at age 68 years, were included. Logistic regression was used to estimate associations between UUI and earlier life vascular risk factors including: lipid status, diabetes, hypertension, BMI, previous stroke or transient ischaemic attack (TIA) diagnosis; adjusting for smoking status, physical activity, co‐presentation of stress UI symptoms, educational attainment; and in women only, type of menopause, age at period cessation, and use of hormone replacement therapy (HRT).

**Results:**

UUI was reported by 12% of men and 19% of women at age 68 years. Female sex, previous stroke or TIA diagnosis, increased BMI and hypertension (in men only) at age 60–64 years were independent risk factors for UUI. Female sex, increased BMI, and a previous diagnosis of stroke/TIA increased the relative risk of more severe UUI symptoms. Type and timing of menopause and HRT use did not alter the estimated associations between UUI and vascular risk factors in women.

**Conclusion:**

Multifactorial mechanisms lead to UUI and vascular risk factors may contribute to the pathogenesis of bladder overactivity in addition to higher BMI. Severe UUI appears to be a distinct presentation with more specific contributory mechanisms than milder UUI.

## Introduction

Urgency urinary incontinence (UUI) is involuntary loss of urine associated with urgency (a sudden compelling desire to pass urine that is difficult to defer) 1, commonly presenting with urinary frequency and nocturia as part of the overactive bladder (OAB) syndrome. UUI is common: prevalence of 30% has previously been reported for those aged >65 years 2, 3. The actual prevalence of UUI is likely to be even higher, as many older people may be too embarrassed to report symptoms 4, 5. Greater frequency or severity of UUI symptoms are associated with worse quality of life 2, 6, 7, 8 and increased risk of depression 9, negatively impacting social interactions, relationships, and self‐esteem 10. The cost of OAB syndrome in the UK alone is currently estimated at £800 million/year, with a further 22% increase anticipated by 2020 as a result of medications, increased need for social care, and higher risk of admission to a nursing home 11.

The multifactorial causes of UUI are reflected in the wide range of risk factors identified in cross‐sectional and longitudinal studies. These include increasing age 12, diagnosis of depression 13, alcohol intake 14, and limitations in physical capability 15. In addition, increased body mass index (BMI) has been consistently associated with all types of UI, including UUI in a number of cohorts 16, 17, 18. It is understood that central adiposity increases intra‐abdominal and bladder pressure in stress UI (SUI) and to a lesser extent in UUI 3. However, increased BMI is also commonly associated with vascular risk factors, such as insulin resistance and glucose intolerance, dyslipidaemia and hypertension, as part of the metabolic syndrome 19. As such, vascular mechanisms may contribute to UUI pathophysiology at the peripheral and central nervous systems, as well as directly on the detrusor muscle and pelvic soft tissue.

Although UUI impacts a significant proportion of both sexes in the population, risk factors for UUI have mostly been explored in cross‐sectional samples of women 17, 18, 20, 21, 22. In the present study, we investigated vascular risk factors assessed at age 60–64 years and UUI subsequently ascertained in men and women at age 68 years within a birth cohort, taking account of a number of clinical and socio‐behavioural factors. We addressed four main questions:

1. What is the prevalence of male and female UI subtypes at age 68 years?
2. Do vascular risk factors contribute to male and female UUI in addition to the effects of raised BMI?
3. Is there evidence for a central neurological contribution to UUI, through the additional contribution of a stroke/TIA diagnosis over and above vascular risk factors in general?
4. Are the same vascular risk factors observed in association with severe and mild UUI?

## Subjects and Methods

The MRC National Survey for Health and Development (NSHD) is the oldest British birth cohort study, following a sample of 5 362 male and female participants, born in 1 week in March 1946. During the 24th data collection in 2014–2015, 2 942 participants in the target sample living in England, Scotland and Wales were contacted and 2 453 (83.4%) returned a postal questionnaire. The target sample did not include participants no longer in the study (*n* = 2 420): 957 (17.8% of the original sample) had already died, 620 (11.6%) had previously withdrawn from the study, 448 (8.3%) had emigrated and had no contact with the study, and 395 (7.4%) had been untraceable for >5 years 23. The 2 294 participants (93.5% of 2 453 who had returned a postal questionnaire), whose symptoms reported on the postal questionnaire corresponded to a recognised UI syndrome were used as the maximum sample for analysis. Of these, 1 753 (76.4%) men and women had full covariate data (detailed below), and comprised the complete sample.

### Urinary Leakage

Questions on urinary leakage were based on the International Consultation on Incontinence Questionnaire Short Form (ICIQ‐SF) 24: ‘1. How often do you leak urine?’, ‘2. How much urine do you usually leak?’, ‘3. How much does leaking urine interfere with your everyday life, on a scale of 0 to 10?’.

Urinary symptoms were categorised into those with (i) UUI only; (ii) SUI only; (iii) mixed UI (MUI). Participants with UUI were defined as those who reported urine leakage ‘before you can get to the toilet’ or ‘when you are asleep’. Participants with SUI were defined by urine leakage ‘when you cough or sneeze’ or ‘when you are physically active or exercising’. MUI was defined as responses with combination of SUI and UUI. A severity score was calculated from the ICIQ‐SF using the sum of question 1, question 2 score multiplied by two and question 3, as recommended 24. A severity score was not calculated for 14 participants who did not respond to all three components. UI severity was defined as: no UI (score of 0), mild UI (3–5), and more severe UI (≥6).

### Vascular Exposures

At age 60–64 years, information was collected by a research nurse at a home or clinic visit 25 on vascular risk factors typically recognised as a component of the metabolic syndrome 19: lipid status, diabetes, hypertension, BMI, and waist circumference. Hypertension was defined as a doctor diagnosis of hypertension, regular prescription of an anti‐hypertensive, or systolic blood pressure of >160 mmHg or diastolic blood pressure >100 mmHg (taken from two readings) 26. Participants reported doctor‐diagnosed type 1 or 2 diabetes mellitus. During the home or clinic visits, waist circumference, height and weight were measured by standardised protocols, and BMI was calculated (kg/m^2^). A fasting blood sample was also taken during this home visit. Lipid status was defined according to whether the participant was hypercholesterolaemic (total cholesterol >6 mm) and/or if a cholesterol‐lowering medication was prescribed. At age 68 years, participants reported any previous diagnosis of stroke/transient ischaemic attack (TIA) ascertained by a doctor.

### Other Covariates

Other covariates selected included: smoking status (defined as current smoker, ex‐smoker, or lifelong non‐smoker, validated against reports at earlier ages); co‐presentation of SUI symptoms at age 68 years; physical activity at age 60–64 years, and educational attainment by the age of 26 years. Educational attainment was categorised into: less than ordinary secondary level; ‘O’ levels; advanced secondary level (‘A’ level); and higher. Participants were asked how many times in the last 4 weeks they had taken part in sports or vigorous activities, categorised as inactive (no episodes), less active (1–4 exercise episodes/month) and more active (≥5 exercise episodes/month). In women, we also accounted for type of menopause (natural menopause, bilateral oophorectomy or hysterectomy with at least one conserved ovary), age at period cessation, and ever‐use of hormone replacement therapy (HRT) 27.

### Statistical Analysis

First, using the maximum sample, the proportion of men and women with UUI at age 68 years by each vascular risk factor and covariate was described. Logistic regression was used to assess the strength of the associations for men and women separately, testing for any sex interactions. Second, we repeated the logistic regressions in 1 762 men and women with complete covariate data, and made a series of three adjustments: (i) for female sex, SUI, and previous diagnosis of stroke/TIA; (ii) additionally for vascular risk factors; and (iii) additionally for all other covariates. Analyses adjusted for BMI per standard deviation (sd) rather than waist circumference as the measure of adiposity; using both would have resulted in collinearity. Third, we estimated multinomial logistic regression models to compare risk factor profiles between those with severe and mild UUI symptoms. Fourth, we estimated logistic regression models to investigate the associations of type and timing of menopause and HRT use on UUI in women, to test whether any observed associations between UUI and vascular risk factors could be accounted for by menopause variables.

## Results

Of 2 294 participants, 825 reported symptoms at age 68 years consistent with a recognised UI subtype. For men, 15% reported UI (UUI 12%, SUI 1.5%, MUI 1%); for women, 54% reported UI (UUI 19% SUI 21% MUI 14%), demonstrating a sex difference (*P* < 0.01). In men, 7% with an UI subtype had severe symptoms, compared with 18% of women.

Co‐presentation of SUI was associated with UUI in both sexes, but was stronger in men than women (men = odds ratio [OR] 5.3, 95% CI: 2.4–11.4; women = OR 1.6, 95% CI: 1.2–2.0; Table 1). Those diagnosed with a previous stroke or TIA reported more UUI (26.3% vs 12.5% for men [OR 2.6, 95% CI: 1.4–4.8]; 47.5% vs 32.6% for women [OR 1.9, 95% CI: 1.0–3.6]). A diagnosis of hypertension was associated with an increased UUI risk in men and women; diabetes was associated with an increased UUI risk in men but not in women (sex interactions with hypertension *P* = 0.49, with diabetes, *P* = 0.03). Raised BMI was associated with increased UUI risk in men and women. No associations between physical activity and UUI were evident.

**Table 1:** Prevalence of UUI and SUI, and ORs for risk factors of UUI in maximum sample of male and female participants in the NSHD cohort

Variable	Female					Male							*P* value for interaction
	*N*	Any UUI at age 68 years, *n* (%)	OR	95% CI	*P*	*N*	Any UUI at age 68 years, *n* (%)	OR	95% CI	*P*			
Any UUI symptoms at age 68 years	1 235	413 (33.4)					1 059	140 (13.2)					
Any SUI symptoms at age 68 years	1 261		**1.6**	**1.2**	**2.0**	**<0.01**	1 156		**5.3**	**2.4**	**11.4**	**<0.01**	**<0.01**
Yes		173 (40.3)						12 (42.9)					
No		240 (28.85)						128 (12.4)					
Any Stroke/TIA diagnosis by age 68 years	1 102		**1.9**	**1.0**	**3.5**	**0.05**	966		**2.5**	**1.3**	**4.7**	**<0.01**	0.52
Yes		19 (47.5)						15 (26.3)					
No		340 (32.6)						104 (12.5)					
Hypertension at age 60–64 years	1 019		**1.3**	**1.0**	**1.7**	**0.05**	836		**1.6**	**1.0**	**2.3**	**0.03**	0.49
Yes		133 (37.9)						55 (15.07)					
No		218 (62.1)						56 (10.3)					
Diabetes by age 60–64 years	1 107		1.0	0.6	1.7	0.89	961		**2.3**	**1.3**	**4.1**	**<0.01**	0.03
Yes		23 (33.8)						18 (24.3)					
No		336 (32.34)						100 (11.3)					
Lipid and statin status at age 60–64 years	1 152					0.45	1 024					0.17	
Normal LDLs		185 (34.58)	Ref					64 (11.49)	Ref				
No statins, raised LDLs		138 (30.8)	0.8	0.6	1.1			24 (11.4)	0.9	0.5	1.5		
Prescribed statins, normal LDLs		56 (33.1)	0.9	0.6	1.4			37 (15.68)	1.4	0.9	2.2		
BMI quintile	1 028					**0.03**	912					**<0.01**	
20%			Ref					16.0	Ref				
40%			1.2	0.8	1.9			23.0	**1.2**	0.6	2.4		
60%			1.3	0.8	1.9			13.0	**0.6**	0.3	1.3		
80%			1.6	1.1	2.5			29.0	**1.4**	0.7	2.6		
100%			1.8	1.2	2.7			30.0	**2.2**	1.1	4.2		
Waist circumference quintile	1 026					**<0.01**	910					0.02	
20%			Ref					8.0	Ref				
40%			1.4	1.0	2.0			18.0	0.8	0.3	2.0		
60%			1.2	0.8	1.8			22.0	0.8	0.3	1.8		
80%			1.5	1.0	2.2			20.0	0.6	0.2	1.4		
100%			2.1	1.4	3.2			43.0	1.4	0.6	3.2		
Smoking status at age 60–64 years	1 082					0.07	954					0.60	
Current	116	32 (26.67)	Ref					10 (9.17)	Ref				
Ex‐smoker	566	205 (35.53)	1.5	1.0	2.4			71 (12.54)	1.4	0.7	2.8		
Never	380	115 (29.87)	1.2	0.7	1.9			35 (12.54)	1.4	0.7	3.0		
Educational attainment by age 26 years	1 261					**<0.01**	1 156					0.89	
<O Levels	480	134 (27.92)	Ref					52 (12.41)	Ref				
O Levels	230	103 (32.19)	**1.2**	0.9	1.7			18 (10.98)	0.9	0.5	1.5		
≥A Levels	461	176 (38.18)	**1.6**	1.2	2.1			70 (12.22)	1.0	0.7	1.4		
Exercise at age 68 years	1 248					0.19	1 143					0.80	
Inactive	748	231 (30.88)	Ref					86 (12.45)	Ref				
Less active (1–4/week)	166	55 (33.13)	1.1	0.8	1.6			15 (12.1)	1.0	0.5	1.7		
More active (>5/week)	334	122 (36.53)	1.3	1.0	1.7			36 (10.98)	0.9	0.6	1.3		

LDLs**,** low‐density lipoproteins. Bold highlights significant findings.

Univariate models in those with complete data confirmed that being female, SUI symptoms, a previous stroke or TIA diagnosis, increased BMI, hypertension and diabetes (in men only) continued to be associated with higher odds of UUI (Table 2, model 1). There were no associations between smoking status, educational attainment or physical activity and UUI. After adjusting for sex, SUI and any stroke or TIA diagnosis the ORs for the other vascular factors were attenuated but independent associations with BMI and hypertension remained (model 2). In the fully adjusted model, being female (OR 4.12, 95% CI: 2.49–6.82; *P* ≤ 0.01), co‐presentation of SUI (OR 1.8, 95% CI: 1.36–2.37; *P* ≤ 0.01), having had a stroke or TIA diagnosis (OR 1.99, 95% CI: 1.14–3.49, *P* < 0.01) and increased BMI (OR 1.19 per sd, 95% CI: 1.05–1.34; *P* = 0.01) were independent risk factors for UUI.

**Table 2:** ORs for UUI risk factors in complete sample of 1 766 participants from the NSHD birth cohort with complete data for vascular risk factors

	Univariate				Adjusted for sex, SUI and stroke				Adjusted for sex, SUI, stroke and vascular risk factors				Adjusted for all covariates			
*N* = 1 753	OR	95% CI		*P*	OR	95% CI		*P*	OR	95% CI		*P*	OR	95% CI		*P*
Sex	**3.70**	**2.88**	**4.75**	**<0.01**									**4.12**	**2.49**	**6.82**	**<0.01**
Any SUI symptoms at age 68 years	**2.98**	**2.32**	**3.83**	**<0.01**					**1.82**	**1.38**	**2.40**	**<0.01**	**1.80**	**1.36**	**2.37**	**<0.01**
Any stroke/TIA diagnosis by age 68 years	**1.82**	**1.09**	**3.03**	**0.02**					**2.02**	**1.15**	**3.52**	**0.01**	**1.99**	**1.14**	**3.49**	**<0.01**
Hypertension at age 60–64 years				**0.03**				**0.02**				0.12				0.12
Men	**1.52**	**1.00**	**2.33**		**1.47**	**0.96**	**2.26**		1.36	0.88	2.09		1.36	0.88	2.10	
Women	**1.39**	**1.04**	**1.84**		**1.29**	**0.97**	**1.72**		1.17	0.87	1.58		1.17	0.87	1.58	
Diabetes by age 60–64 years				0.48				0.52				0.95				0.95^a^
Men	**1.94**	**0.99**	**3.81**		1.81	0.92	3.57		1.63	0.81	3.26		1.61	0.81	3.23	
Women	1.02	0.56	1.86		0.85	0.46	1.58		0.68	0.35	1.30		0.68	0.36	1.31	
Lipid and statin status at age 60–64 years				1.00				0.16			0.31					0.30
Normal LDLs	Ref				Ref				Ref				Ref			
No statins, raised LDLs	1.01	0.79	1.29		0.79	0.61	1.03		0.82	0.63			0.82	0.63	1.07	
Prescribed statins, normal LDLs	0.99	0.73	1.35		1.02	0.73	1.42		0.88	0.62			0.88	0.62	1.26	
BMI (per sd) at age 60–64 years	**1.26**	**1.12**	**1.40**	**<0.01**	**1.22**	**1.09**	**1.37**	**<0.01**	**1.19**	**1.06**	**1.34**	**<0.01**	**1.19**	**1.05**	**1.34**	**0.01**
Smoking status at age 60–64 years				0.61				0.31								0.40
Current	Ref				Ref								Ref			
Ex‐smoker	1.20	0.82	1.76		1.29	0.87	1.92						1.26	0.85	1.88	
Never	1.12	0.75	1.68		1.11	0.73	1.69						1.10	0.72	1.68	

LDLs, low‐density lipoproteins.

a *P* value for sex interaction <0.1. Bold highlights significant findings.

A previous diagnosis of stroke/TIA increased the relative risk of severe UUI symptoms (relative risk ratio [RRR] 3.65, 95% CI: 1.87–7.1); no corresponding association was seen with mild UUI. Increased BMI and being female were risk factors for both mild and severe UUI (Table 3). There were no sex interactions in these multivariate models. Type and timing of menopause and HRT use did not alter the estimated associations between UUI and vascular risk factors in women (Table 4).

**Table 3:** Multinomial regression analyses of RRRs in mild and severe UUI in 1 761 participants from the NSHD with complete data for vascular risk factors and UI severity scores

	Adjusted for all covariates							
	Mild UUI				Severe UUI			
*N* = 1 748	RRR	95% CI		*P*	RRR	95% CI		*P*
Female sex	**3.51**	**2.58**	**4.77**	**<0.01**	**4.96**	**3.25**	**7.58**	**<0.01**
Any stroke/TIA diagnosis by age 68 years	1.08	0.49	2.40	0.84	**3.65**	**1.87**	**7.10**	**<0.01**
Hypertension at age 60–64 years	1.21	0.90	1.63	0.21	1.31	0.89	1.91	0.17
Diabetes by age 60–64 years	0.96	0.52	1.78	0.89	1.18	0.60	2.34	0.63
Lipid and statin status at 60–64 years								
No statins, normal LDLs	0.95	0.70	1.28	0.72				
No statins, raised LDLs	0.93	0.61	1.42	0.74	0.66	0.44	0.99	0.05
Prescribed statins, normal LDLs					0.82	0.49	1.38	0.46
BMI at age 60–64 years	**1.13**	**0.98**	**1.30**	**0.09**	**1.32**	**1.12**	**1.57**	**<0.01**
Smoking status at age 60–64 years								
Current	Ref				Ref			
Ex‐smoker	1.34	0.82	2.20	0.24	1.19	0.67	2.13	0.55
Never	1.37	0.82	2.29	0.23	0.71	0.37	1.35	0.30

LDLs, low‐density lipoproteins. Bold highlights significant findings.

**Table 4:** ORs for UUI in female NSHD participants with complete data for women's health and vascular risk variables

Variable, *n* = 752	Univariate (complete *n*)				Adjusted for all covariates			
	OR	95% CI		*P*	OR	95% CI		*P*
Type of menopause								
Natural	Ref			0.29	Ref			
Hysterectomy	1.25	0.83	1.88		1.65	0.94	2.88	0.08
Bilateral oophorectomy	1.38	0.85	2.26		1.54	0.89	2.64	0.12
Period cessation age (years)								
Natural	1.30	0.80	2.09	0.29	1.28	0.79	2.08	0.32
Hysterectomy	1.17	0.61	2.24	0.64	1.16	0.60	2.25	0.67
Bilateral Oophorectomy	2.09	0.81	5.39	0.13	2.17	0.83	5.67	0.11
Ever used HRT								
Natural	0.92	0.65	1.32	0.59	0.89	0.62	1.28	0.53
Hysterectomy	0.66	0.27	1.63	0.48	0.74	0.29	1.88	0.53
Bilateral oophorectomy	3.66	0.41	32.95	0.24	4.83	0.47	49.36	0.18
Any SUI symptoms at age 68 years	**1.60**	**1.17**	**2.19**	**<0.01**	**1.58**	**1.14**	**2.17**	**0.01**
Any stroke/TIA diagnosis by age 68 years	1.79	0.78	4.11	0.17	1.60	0.67	3.82	0.29
Hypertension at age 60–64 years	1.26	0.92	1.73	0.15	1.13	0.80	1.59	0.48
Diabetes by age 60–64 years	1.00	0.50	1.98	0.99	0.76	0.35	1.64	0.48
Lipid and statin status at age 60–64 years				0.37				0.33
Normal LDLs	Ref				Ref			
No statins, raised LDLs	0.79	0.57	1.10		0.81	0.58	1.13	
Prescribed statins, normal LDLs	0.86	0.54	1.38		0.74	0.44	1.25	
BMI at age 60–64 years	**1.17**	**1.02**	**1.34**	**0.02**	1.14	0.99	1.33	0.07

LDLs, low‐density lipoproteins. Bold highlights significant findings.

## Discussion

In a large representative British population cohort at the age of 68 years, the prevalence of UI was 15% in men (where UUI was the most common subtype), and 54% in women (with similar proportions of UUI and SUI subtypes). Female sex, a previous diagnosis of stroke/TIA, increased BMI, and SUI were associated with UUI symptoms. Stronger associations were found between these risk factors and UUI if severe UUI was reported. For those with milder symptoms, the associations were weaker, except for the negative association with educational attainment. In women, no associations were found between UUI symptoms and menopause or HRT use. Taken together, these results suggest that vascular risk factors, in particular hypertension, may contribute towards UUI pathophysiology in addition to previous stroke/TIA, raised BMI, female sex and co‐presentation with SUI.

A strength of these analyses was the prospective ascertainment of all variables. We used the ICIQ‐SF, a structured, validated scale with a specific component evaluating impact on daily life. A limitation is the lack a definite operationalisation of UI subtypes in ICIQ‐SF, excluding a small number of participants reporting only atypical symptoms. Also, the severity of UI was measured using self‐reported symptom impact, without objective urodynamic measures. Third, vascular covariates could not be further characterised by cumulative exposure, such as duration since first diagnosis, introducing a dose‐dependent element to the associations. Lastly, the definition of previous TIA/stroke did not differentiate between temporary or permanent neurological deficits, size of lesion or neuroanatomical location of stroke.

Whilst the estimated prevalence of female UUI at the age of 68 years was similar to other studies with samples of similar ages 21, 22, UUI prevalence in men was higher than that reported (11.7% for men aged >65 years, 95% CI: 9.27–14.14%) in a recent pooled analysis of men aged >65 years 28. The increased prevalence of UUI in both men and women from age 53–68 years is consistent with previous single and multicentre cross‐sectional studies reporting increasing age as a major risk factor for UUI 15, 22, 29. The prevalence of vascular risk factors in the NSHD was comparable to published literature: the prevalence of diabetes and hypertension at age 60–64 years were 6.9% and 38%, respectively. In addition, 4.8% of participants had been diagnosed with a stroke or reported TIA symptoms by the age of 68 years. These are comparable to published literature: the prevalence estimates of hypertension between 55 and 64 years was 45% according to the Health Survey for England statistics report from 2012 30; 6.5% for diabetes at age 55–64 years 31; and 3% for men and 2% for women for prevalence of stroke between 55 and 64 years using the British Heart Foundation stroke statistics 2009 32. The remaining discrepancies are likely explained by the 100% White Caucasian demographics of the NSHD cohort, whilst other analyses included participants of Afro‐Caribbean and South Asian ethnicities, where prevalence of hypertension and diabetes respective are generally higher.

Our present findings suggest severe UUI to be a distinct entity compared to mild UUI at this age, with more specific risk factors. First, a syndrome of mild UUI possibly reflects increased recognition of UUI symptoms as atypical for normal ageing, with relatively less contribution of specific vascular risk factors. In contrast, severe UUI is strongly associated with previous stroke/TIA, which may result from a greater contribution of vascular disease and central nervous pathology. Patients with central lesions, such as strokes and multiple sclerosis, commonly report UUI as a complication. Structurally, subcortical white matter lesions, a marker of chronic vascular burden, are associated with increased symptoms of detrusor overactivity 33. However, central pathology can contribute to UUI even in the absence of overt signs of neurological disease 34: functional MRI studies involving patients with UUI without diagnosed neurological pathology have associated UUI symptoms with abnormal activation patterns in the prefrontal and anterior cingulate cortex, insula, basal ganglia, and cerebellum 34.

**Figure 1:**
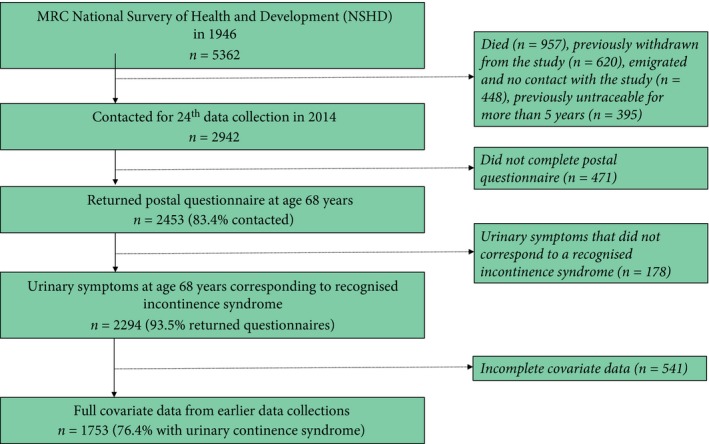
Flow diagram of study.

Whilst the present study corroborates previous findings that BMI plays a central role in the aetiology of UUI 16, 17, our results also support the emerging view that obesity contributes to UUI as part of a wider metabolic syndrome. This is shown in men but not so in women. The association between type 2 diabetes and male UUI in our present study is consistent with previous studies linking UUI with markers of poor glycaemic control, including diagnosis of type 2 diabetes mellitus 35, higher glycated haemoglobin (HbA1c) in patients with type 2 diabetes 36, raised serum insulin, and increased homeostatic model assessment of insulin resistance (HOMA‐IR) 20. Our association of hypertension and UUI adds to a previous study linking hypertension with OAB symptoms in a female cohort 14 (Figure 1).

The present study provides prevalence of UI subtypes for men and women within a representative large cohort in their late 60s. We demonstrate that severe UUI is a distinct disease entity, whilst those with milder UUI may represent a broad range of contributory mechanisms. Our present findings suggest that multifactorial mechanisms lead to UUI and that vascular risk factors are additionally associated, potentially playing a role in the development of pathological bladder overactivity.

## Conflict of Interests

The authors declare no conflicts of interest.

**Abbreviations**
(S)(M)(U)UI: (stress) (mixed) (urgency) urinary incontinence
BMI: body mass index
HRT: hormone replacement therapy
ICIQ‐SF: International Consultation on Incontinence Questionnaire Short Form
NSHD: National Survey for Health and Development
OAB: overactive bladder
OR: odds ratio
RRR: relative risk ratio
TIA: transient ischaemic attack
